# Supplementary material for: The Potential of Edible and Medicinal Resource Polysaccharides for Prevention and Treatment of Neurodegenerative Diseases
Source: Biomolecules. 2023 May 22;13(5):873. doi: 10.3390/biom13050873 (PMC10216080; doi:10.3390/biom13050873)
Supplement: Supplementary file 1 [file biomolecules-13-00873-s001.zip › biomolecules-2276514-supplementary.pdf]

Supplementary Materials

The Potential of Edible and Medicinal Resources Polysaccharides for Prevention and Treatment of Neurodegenerative Diseases

Qingxia Gan [1,2], Yugang Ding [1,2], Maoyao Peng[1,2], Linlin Chen[1,2], Jijing Dong[1,2], Jiaxi hu[1], Yuntong Ma [1,2,*]

[1] College of Pharmacy, Chengdu University of Traditional Chinese Medicine, No.1166, Liutai Road, Wenjiang District, Chengdu, 611137, China
[2] State Key Laboratory of Traditional Chinese Medicine Processing Technology, State Administration of Traditional Chinese Medicine, No.1166, Liutai Road, Wenjiang District, Chengdu, 611137, China
* Correspondence: mayuntong@cdutcm.edu.cn

Table S1. Summary of the role of polysaccharides in the regulation of neurodegenerative diseases.

	Sources of polysaccharides	Characterization	Models	Doses and time of polysaccharides	Biological activity	References
**1.**	***Abelmoschus moschatus* polysaccharides (OP)**	Man, Rha, Glc, Gal, and Ara	1.0 mg/mL of 2 μL Aβ1–42 induced AD model mice,1 time	300, 600 mg/kg (OPL) for 8 weeks	↓Cognitive impairment<br>↑PI3K and AKT, pERK1/2<br>↓GSK3β<br>↑PI3K/AKT and ERK pathways	[20]
**2.**	***Inonotus obliquus* polysaccharides (IOP)**	111.9 kDa, no nucleic acids or proteins	25mM L-Glu-induced HT22 cell for 24h<br>APP/PS1 mice	Pre-treated with 5 or 10 μg/mL for 3 h<br>25 or 50 mg/kg for 8 weeks	↑Spatial learning and memory<br>↓Oxidative stress, apoptosis<br>↓Deposition of Aβ1–42 and P-Tau<br>↑Bcl-2, Nrf2, HO-1, SOD-1, GCLC<br>↓Bax, Keap1<br>↑Nrf2 pathway	[22]
**3.**	***Ganoderma lucidum* polysaccharides (GLP)**	15.0 kDa	APP/PS1 transgenic mice<br>1 ng/mL EGF and 1 ng/mL bFGF induced neural progenitor cell for 24 h	30 and 200 mg/kg for 90 days<br>After-treated with 300 mg/mL for 24h	↑Cognitive Function<br>↑NPC Proliferation<br>↑FGFR1, p-ERK, p-AKT<br>↑ERK and AKT pathway	[24]
**4.**	***Momordica Charantia* polysaccharides （MCP）**	85-100 kDa<br>Ara, Xyl, Gal, and Rha (1.01 : 1.13 : 4.17 : 1.67)	C57B/6 male mice were intraperitoneally injected 25 mg/kg MPTP for 7 days<br>1mM MPP+ induced SK-N-SH cells for 24 hours	After-treated with 100 mg/kg were given for 7 days<br>After-treated with 80 μg/mL for 24 hours	↑Cell viability<br>↓Dyskinesia<br>↓Inflammation, Oxidative Stress, and Apoptosis<br>↑DA, DOPAC, HVA, GSH, SOD, Bcl-2<br>↓5-HT, 5-HIAA, TNF-α, IL-1β, MDA, Cytochrome C, Bax, cleaved Caspase-3 in the brain tissue<br>↑TH in the striatum<br>↓TLR4, MyD88, p-p65	[25]

				↓TLR4/MyD88/NF-κB pathway	
		HEK293 cells were transfected with EGFP-exon-1 htt-20Q and EGFP-exon-1 htt-160Q<br>HD-N171-82Q-81 (also known as B6C3-Tg (HD82Gln)81Dbo/J Tg) mice	After-treated with 9.6 μg/μL LBP for 24 h.<br>40 mg/kg body weight per day for10 weeks	↑Life span, body weight, motor functions<br>↓mHtt aggregates, caspase-3<br>↑p-AKT-Ser473<br>↑AKT pathway	[26]
**5.**	***Lycium barbarum* polysaccharides (LBP)**	30 mg/kg/d MPTP induced male C57BL/6 mice for 5 days	After-treated with 100 and 200 mg/kg for 16 days	↑Spontaneity, coordination, learning and memory abilities<br>↓aggregation of α-syn<br>↓Dopaminergic Neurodegeneration, LC3I/Π and Beclin<br>↑SOD2、CAT and GPX1<br>↑p-AKT and mTOR<br>↑PTEN/AKT/mTOR pathway	[44]
	Man, Glc, Gal, Xyl and Ara (6.52: 78.12: 8.85: 1.81: 4.69.)	APP/PS1 transgenic mice<br>HEK293/APPswe cells	Oral administration of 50 mg/kg for 3 months<br>300 μg/ml for 24 hours	↑Cognitive functions<br>↑Neurogenesis<br>↓Synaptic dysfunction<br>↓Aβ levels and amyloid plaque burden	[104]
		HTM cells were exposed to 200 μM H_2O_2 for 24 h	Pre-treated with 100, 200,300, 400 and 500 μg/mL for 1 h	↓Oxidative damage, apoptosis<br>↑ miR-4295<br>↓Cleaved-caspase-3/-9 and ROS<br>↑PI3K/AKT and ERK signaling pathways	[121]
		N2a cells 3.75μM TMT for 24h	Pre-treated with 300 μg/mL for 24 h	↓Apoptosis and oxidative stress	[122]

					↑Bcl-2, SOD, caspase-3, p-GSK-3β, p-Akt, Shh, Gli1, CyclinD1<br>↓ROS, MDA<br>↑Shh and PI3K/Akt pathways<br>↓Apoptosis and oxidative stress	
		L-Rha, Glc, and erythritol	20 mM L-Glu-induced PC12 cells for 24 h	Pre-treated with 10 and 30 μg/ml alone for 3 h	↑MMP, Bcl-2, p-Akt, and p-ERKs<br>↓ ROS, Bax, caspase-3<br>↑Akt and ERK pathway<br>↓Mitochondrial apoptotic pathway	[123]
			PC12 cells induced by 400 μM H2O2 for 24h	Co-treated with 125-500 μg/ml for 24 h	↓Oxidative stress and apoptosis<br>↓Caspase-3, Caspase -9, ROS,<br>↑Nrf2 and HO-1 mRNA and protein<br>↑Nrf2/HO-1 pathway	[133]
		45.0 kDa;<br>Ara, Gal, Glc and Rha (47.8: 49.8: 1.4: 1.2)	HEK293-APPsw cells	0.6, 1.1, 2.2 μM for 24 h or for 72 h	↓Aβ42 production and aggregation	[134]
			75 μM 6-OHDA induced PC12 cells for 24 h	Pre-treated with 100, 300, 600 μg/mL LBP for 24 h	↓Apoptosis and oxidative stress<br>↓ROS, NO, 3-NT, intracellular free Ca^{2+}, NF-κB, nNOS, iNOS, caspase-3<br>↓ROS-NO pathway	[135]
**6.**	***Codonopsis pilosula* polysaccharides (CPP)**		APP/PS1 mice	100 and 300 mg/kg for one month	↑Cognitive ability<br>↓$A\beta_{1-42}$ deposition	[40]
			N2a and HEK293 cells transfected with APP plasmid	200μg/ml for 24h	↑synaptic plasticity, synaptic proteins (PSD95, synaptotagmin)<br>↓BACE1	

			Injected 2 ml of 6.82×1012v.g./ml AAV2/hTau into the hippocampus of C57/BL6 mice<br>HEK293/tau cells	After-treated with 100, 300 mg/kg for 1 month.<br>After-treated with 50, 100, 200 mg/ml for 48 h.	↓Memory Deficits<br>↑PP2A Activity<br>↓Tau Phosphorylation<br>↑Synaptic Plasticity and Functions<br>↑Synaptotagmin, synaptophysin	[43]
		Man (1.76%), Glc (97.38%), and Ara (0.76%).	1 μM Aβ_{1-40} induced PC12 cells for 24 h	Co-treated with 50 μg/ml for 24 h	↑Cellular Viability<br>↑ATP, MMP, NAD+、NAD+/NADH、SIRT3、SIRT1, PGC-1α<br>↓ROS	[136]
**7.**	***Hericium erinaceus* polysaccharides (PHEB)**	36.1 kDa<br>D-Gal, D- Glc, D-Man and D-GlcA	APP/PS1 mice	25 and 100 mg/kg for six weeks after fed for 24 weeks	↑Cognitive ability<br>↓Inflammation<br>↓Aβ deposition and tau hyperphosphorylation<br>↓ChAT, Ach, MDA, ROS<br>↑AChE, SOD, CAT, GSH-Px<br>↑Nrf2, HO-1, GABBR1, PKA, GluT1, Neurogranin, p-Akt, p-mTOR<br>↓Keap1, CaMK IV, p-CaMK II, ERK 1/2, Ras, P-GluR2<br>↑Akt/ mTOR and Nrf2 pathway	[41]
**8.**	***Schisandra chinensis* (Turcz.) Baill polysaccharides (SCP)**	Ara, Glc, Gal, Man, Ribose, Xyl, GlcA, Rha and Fucose	AD model induced by 10 μg of 2 μg/μL Aβ_{25-35} injection in the rat hippocampal CA1 region, 1 time	After-treated with 38.15 mg kg 1 day for 56 consecutive days	↑Spatial learning and memory ability<br>↓AChE, GSK-3β, NOS, Glu, Asp<br>↓Aβ_{25-35} and p-Tau aggregations<br>↑SOD, 5-HT, NE, DA, Ach, Tau, GABA, Gly	[42]

	Man, Rha, GlcA, Glc, Gal, and Ara	Male KM mice were intracerebroventricular injected with 3μL Aβ1–42 peptide into the left lateral ventricle, 1 time	After-treated with 260 mg/kg for 28 consecutive days (i.g.).	↑Learning memory, cognitive capacity<br>↓Inflammation, activation of astrocytes and microglia<br>↓Aβ deposition<br>↓NF-κB in nuclear, p-p-38, p-JNK, p-ERK, IL-1β,1L-6, TNF- α<br>↑NF-κB and IκB-α in cytosolic<br>↓NF-κB/MAPK pathway	[79]
**9.** ***Angelica sinensis*** **polysaccharides (ASP)**		All rats were injected with 5μL of 4 μg/μL Aβ25–35 into bilateral CA1 subregion,1 time	After-treated with 50 mg/kg for 4 weeks	↑Spatial learning and memory<br>↓Inflammatory, apoptosis<br>↑Ach, chAT, SOD, CAT, BDNF, TrkB, p-Akt, p-CREB<br>↓MDA, AchE, IL-1β, IL-6, TNF-α<br>↑BDNF/TrkB/CREB pathway.	[45]
		Male Nestin-GFP transgenic mice were subcutaneously injected with 200 mg/kg D-Gal for 42 days	140mg/kg ASP, ip, for 27days since the 16th day of D-Gal induction model	↑Cognitive ability<br>↓Oxidative stress, inflammatory<br>↑Na^+-K^+ - ATP, SOD, T-AOC<br>↓MDA, IL-1β, IL-6, TNF-α, ROS, p53 mRNA, p21 mRNA<br>↓P53/p21pathway	[127]
		NSCs Induced by 20mg/mL D-gal for 48h	After-treated with 100 μg/ml for 24h		
**10.** ***Astragalus membranaceus*** **polysaccharides (APS)**	19.5% Ara, 6.4% Rha, 15.6%Gal, 29.5% GalA and 29.0%Glc.	*C. elegans* strain AM141	0.25, 1.0, 2.5, 5mg/ml for 24, 48, 72, 96 h	↓polyQ aggregates	[46]
		C. elegans strain HA759	0.25, 1.0, 2.5mg/ml for 3 days at 15°C	↓Neurotoxicity	
		C. elegans strain N2, AM141, HA759	1.0, 2.5mg/ml until death at 15°C	↑Lifespan	

	C. elegans strain N2, AM141, HA759	1 mg/ml for 3 days	↑DAF-16/FOXO<br>↓Gene scl-20	
	BV2 microglial cells induced by 50 ng/ml LPS for 24 h	Pre-treated with 0, 50, 100, or 200 μg/ml for 1 h	↓Inflammation<br>↓iNOS mRNA, COX-2 mRNA, TNF-α mRNA, IL-1β mRNA, TNF-α, IL-1β, NO, PGE2<br>↓NF-κB and PKB pathway	[83]
	100 μM 6-OHDA in PC12 cells for further 24 h	Co-treated with 0,50, 100 and 200 μM for 24 h	↑Autophagy<br>↑LC3-I→LC3-II<br>↓p-mTOR and p-Akt mRNA and protein<br>↑PTEN mRNA and protein<br>↓PI3K/AKT/mTOR pathway	[100]
	APP/PS1 mice	200 mg/kg b.w./d, for 2 months	↓Oxidative stress and apoptosis<br>↓Deposition of Aβ40 and Aβ42<br>↑Nrf2 in the nucleus, SOD, GSH-Px<br>↓Nrf2 in the cytoplasm, Keap1, MDA<br>↑Nrf2 pathway	[117]
5.14 kDa; Myo-inositol: fructose: sorbitol: Glc (1:1.4:2.1:13.7:91.5)	APP/PS1 mice	500 mg/kg twice per day for 7 weeks	↑Cognitive ability<br>↓Neuroinflammation<br>↓Activation of Astrocytes and Microglia	[137]
19.2% Ara, 6.9% Rha, 16.0% Gal, 28.8% Glc, and 29.1% GalA.	50mM 6-OHDA induced transgenic *C. elegans* strain BZ555 for 1 h at 20 °C.	0.5, 1, 2,4mg/mL for 72h<br>2.0mg/mL until death	↓Food-Sensing deficit, degeneration of dopaminergic neurons<br>↑Lifespan	[138]

			2.0mg/mL for 72h	↓Oxidative stress<br>↓ROS, MDA, Gene egl-1<br>↑SOD, GPx, AChE	
**11.** ***Trametes versicolor*** **Polysaccharides (PSK)**		APP/PS1 mice	2 mg/mouse, 3 times/week for 6 months (i.p.)	↓Cognitive deficits<br>↓Activation of astrocytes and microglia<br>↓Aβ40, total Aβ, Aβ42 in brain, serum Aβ42<br>↓pro-inflammatory cytokines TNF-α and IL-6 in brain homogenates, caspase-3<br>↓Tau Phosphorylation in the hippocampus	[54]
12. Fomes officinalis **Ames polysaccharides (FOAP)**	Ara, Xyl, Man, Glc, and Gal	40μM Aβ25–35 induced PC12 cells for 48 h	After-treated with 50, 100 and 200 lg/mL for 2 h	↓Apoptosis, oxidative stress<br>↑Cell viability<br>↓LDH, ROS, MDA<br>↑Bcl-2/Bax, SOD, MMP, ATP	[67]
13. Epimedium brevicornum **polysaccharides (EbPS-A1)**	56.7% GalA, 19.4%Gal and 16.1%Rha but also 5.9%Ara and 2.0% GlcA	*C. elegans* HA759	0.5, 1, 2, 4 mg/mL for 3 days at 20°C.	↓Chemosensory dysfunction	[68]
			2 mg/mL at 20°C for 3 days	↓Free radicals, ROS, MDA<br>↑SOD	
**14.** ***Saccharina japonica*** **sulfated hetero-polysaccharides (UF)**	Fucose: Uronic Acid: Sulfate (19.12:14.25:21.2)	100 µM H2O2-induced SH-SY5Y for 24 h.	Co-treated with 100, 500, 800 µg/mL for 24 h	↓Apoptotic<br>↑p-PI3K, p-Akt, p-NGF<br>↓Bcl-2, Bad/bax, caspase-3, caspase-8, caspase-9, GSK3β, Cytc, p53<br>↑PI3K/Akt Pathway	[53]

15. ***Laminaria japonica* polysaccharides (Fucoidan)**		Subcutaneous injection of 1.5 mg/kg/d rotenone induced Male Sprague–Dawley rats 5 times a week for 4 weeks	35, 70, 140 mg/kg/d started 10 days before rotenone induction, for 38 days	↓Behavioral deficits<br>↓Oxidative stress<br>↑TH-positive neurons and striatal TH-positive fibers, mitochondrial respiratory capacity<br>↑DA, DOPAC, PGC-1α, NRF2<br>↓MDA, 3-NT, 8-OHdG<br>↑Nrf2 pathway	[69]
	Fucose: Gal (1.0:0.24)	Microglial cells induced by 0.01 μg/ml LPS for 24h	Pre-treated with 31.25, 62.5 and 125μg/ mL for 10 min	↓Inflammation<br>↓NO production, iNOS expression, p-p38 and p-ERK<br>↓p38 and ERK pathway	[112]
		C. elegans GMC101	20, 100, 500 and 2500 ng/ml for 31 h from 16 °C to 25 °C	↓Paralysis rate<br>↓deposition of Aβ protein<br>↓ROS<br>↑hsp16.2 expression	[139]
16. **Fucoidan from *Fucus vesiculosus* (FVF)**	Fucans, Gal,Xyl.	20 mg/kg MPTP induced male C57BL/6 mice	10 and 40 mg/kg for 8days starting from one day before MPTP injection	↓Motor deficits<br>↓Mitochondrial dysfunction<br>↓Neuronal apoptosis<br>↓Dopaminergic neuron<br>↑ATP5F1a protein	[140]
**17.** ***Korean red ginseng* polysaccharides (NFP)**	GalA, Gal, and Ara (139.6:74.7: 63.4)	5XFAD mice	200 mg/kg for 3 weeks	↑Cognitive function<br>↓Inflammation, apoptosis<br>↓Aβ deposits<br>↓Mitochondrial dysfunction<br>↑Neurogenesis	[80]
**18.** ***Chlorella pyrenoidosa* polysaccharides**	Glc (69.14%), Rha (15.36%), Gal (14.29%),	Male C57BL/6 mice with 15 mg/kg MPTP four times at 2h interval (i.p.).	Oral administration 100,200 mg/kg/d was started 11 days before modeling, for 19 days	↓Bradykinesia<br>↓striatal depletion of DA, DOPAC and HVA<br>↑TH gene and nigral TH protein	[81]

	**(CPS)**	Man (1.16%) and Xyl (0.06%).			↓striatal Emr1, TNF-α, IL-1β and IL-6 in serum.<br>↑serum DAO and small intestinal secretory immunoglobulin A S-IgA	
19.	***Bletilla striata* polysaccharides (BSP)**		Aβ fibrils induced N2a cells for 24 h	Co-treated with 1 mg/mL BSP for 24 h	↓Learning memory<br>↑Cell Viability	[82]
			10μM Aβ induced BV-2 cells	Co-treated with 1 mg/mL BSP for 24 h	↓Oxidative stress, Inflammation<br>↓Aβ Fibril Formation, Morphological damage in the hippocampus and cortex	
			100 mg/kg AlCl3 induced SD rats three times every week, for eight weeks	Co-treated with 10 mg/kg/day for eight weeks	↓ROS, TNF-α, IL-6, and IL-10<br>↓BACE1	
**20.**	***Antrodia camphorata* polysaccharides (ACP)**		5 μl of 2 μg/μl 6-OHDA solution was injected unilaterally into the median forebrain bundle at a time	After-treated with 10, 50, 100 mg/kg for 14 days	↑Spontaneity, coordination, learning and memory abilities<br>↓Inflammation<br>↓NLRP3, IL-1β, Caspase 1, and proCaspase 1<br>↓NLRP3 pathway	[84]
			6-OHDA induced MES23.5 for 24 h	Pre-treated with 10, 20, 50mM for 3h		
			5 μl of 2 μg/μl 6-OHDA solution was injected unilaterally into the median forebrain bundle at a time	10, 50, and 100 mg/kg for 7 days.	↓NLRP3, ASC, caspase-1, IL-1β, IL-18, ROS<br>↓ROS-NLRP3 pathway	[85]
21.	***Sparassis crispa* polysaccharides (SCP-1)**	Galactoglucan, main chain contains (1 → 6)-β-D-Glcp,	Oral with AlCl3 (20/kg/d) and subcutaneously injected with D-Gal (120 mg/kg/d) for eight weeks	25 mg/kg/d and 100 mg/kg/d once per day from the fifth week for four weeks	↓learning deficits and defective spatial recognition<br>↓Amyloidogenesis<br>↑Neurotransmitter levels (γ-aminobutyric acid, glutamate, and acetylcholine)	[86]

	(1 → 3)-β-D-Glcp,<br>(1 → 6)-α-D-Galp			↓Inflammatory cytokines<br>↑Reshaped the gut microbiota<br>↑Intestinal barrier function, SCFAs<br>↓TLR4 and NF-κB	
**22.** ***Sparassis crispa*** **polysaccharides (SCWEA)**	75kDa;<br>Rha, Man, and Gal (Gal) 10:2:1	25 mM L-Glu induced PC12 cell for 24 h	Pre-treated 4 μg/mL for 3 h	↓Apoptosis and oxidative stress<br>↓Ca2+, ROS,<br>↑MMP, Bcl-2, Bcl-xL, p-AKT, p-GSK-3<br>↑AKT pathway.<br>↓Mitochondrial pathway	[109]
	18.796 kDa	5 × FAD mice	30 mg/kg per day at 3 months	↑Behaviors related to memory and cognition<br>↓Synaptic loss<br>↓Microglial phagocytosis of Aβ plaques, Aβ1–40 and Aβ1–42<br>↑Reconstructed the gut microbiota composition<br>↓Gut barrier integrity damage, inflammatory responses, and the intestinal Aβ deposition	[87]
23. Polygonatum sibiricum **polysaccharides (PSP)**		30 mg/kg MPTP (i.p.) induced C57BL/6J male mice for five days.	10, 30 mg/kg PSP orally started 30 days before MPTP induction, for 45 days	↑Motor abilities<br>↓Apoptosis, Oxidative Stress，Dopaminergic neurodegeneration	
		500 μM MPP+ induced N2a cells	Pre-treated with1, 10, 100, 200, 400 μg/mL for 24 h	↓ROS<br>↑GSH/GSSG<br>↑p-Akt, p-mTOR, Nrf2, NQO1, HO-1, Gclc, Gclm<br>↑Akt/mTOR and Nrf2 Pathways	[118]
	76 kDa, Gal:Man (12.1:5.4)	20μM $A\beta_{25–35}$-induced neurotoxicity in PC12 cells	Pre-treated with 10, 20, 50, 100 and 200 μg/ml for 24 h	↓Bax/Bcl-2 ratio, caspase-3<br>↑p-Akt<br>↑PI3K/AKT pathways	[124]

**24.** ***Spirulina platensis*** **polysaccharides (PSP1)**		Male C57BL/6J mice (i.p.) with 15 mg/kg MPTP 4 times, at 6-hour intervals	Pre-treated with 200, 400, or 800 mg/kg for 10 days	↓Oxidative stress<br>↑TH and DAT protein and mRNA in the SN pars compacta<br>↑SOD, and GSH-Px	[102]
**25. Edible** ***Dictyophora echinovolvata*** **polysaccharides (DEVP)**		PC12 cells induced by 750mM H2O2 for 24 h	Pre-treated with 10, 30, 100, 250, 500 mM for 2 h	↓Apoptosis and oxidative stress<br>↑Bax, caspases 3, cytochrome c<br>↓ Bcl-2, ROS<br>↓Mitochondrial apoptotic pathway	[107]
**26.** ***Tremella fuciformis*** **polysaccharides （TL04）**	Rha, Man and Glc, 1:5.04:1.87	20 mM L-Glu induced PC12 cells for 12-24 h	Pre-treated 5 and 20 μg TL04 for 3h	↓Apoptosis and oxidative stress<br>↓ROS, caspase 3, Bax, Cyto C, caspase 8, caspase 9 and caspase 3, LDH<br>↑Bcl-2<br>↓Mitochondrial pathway	[108]
**27.** ***Armillaria mellea*** **polysaccharides (AMPS)**		HT22 cells incubated with 25mM of L-Glu for another 24 h	Pretreated with AMPSc at doses of 40 and 80 μg/mL for 3 h	↑Horizontal movements, learning and memory, cognitive abilities<br>↓Apoptosis and oxidative stress<br>↓Aβ_{1-40} and p-Tau aggregations<br>↓MMP, ROS, AchE, ROS<br>↑Ach, ChAT, SOD, GSH-Px, 4-NHE	[110]
**28.** ***Morchella importuna*** **polysaccharides (MIP)**		PC12 cells induced by 0.8mM H2O2 for 6h	Pre-treated with 12.5, 50, 100, 200, 400, 800μg/ml for half an hour	↓Oxidative stress and apoptosis<br>↑SOD, CAT, GSHPx<br>↓ Bcl-2, Bax, caspase-3<br>↑ERK pathway<br>↓NF-κB pathway and the p38-JNK pathway	[113]
29. ***Acorus tatarinowii*** **polysaccharides (ATP)**		1 μg/mL LPS induced proinflammatory BV2 cells for 24 h	Pretreated with 2.5, 5.0, 10 μM for 2 h.	↓NF-κB, TLR4, MyD88, p-PI3K, p-Akt,<br>↓ROS, MMP, TNF-α, IL-1β, IL-6, COX2, and iNOS	[115]

**30.** ***Vitis vinifera L.*** **polysaccharides (VTP)**		10mL of 2mg/mL Aβ25–35 slowly injected into the hippocampal CA1 region (5ml per side) of Sprague Dawley rats,1 time	50,150, 300 mg/kg VTP for 14 days.	↓TLR4-mediated MyD88/NF-κB and PI3K/Akt signaling pathways<br>↑Learning and memory abilities<br>↓Apoptosis, Inflammation<br>↓TNF-α, IL-6 and IL-1β in serum, NF-κBp65 and IkB-a mRNA level<br>↓p-NF-κB-p65, p-IkB-a<br>↓NF-κB pathway	[141]
**31.** ***Taxus chinensis var. mairei Cheng et L.K.Fu (Taxaceae)*** **polysaccharides (PTM)**		150mg/kg/d D-Gal induced C57BL/6 mice (i.p.) for 6weeks<br>BV2 induced by 1M D-gal for 48 h.<br>Balb/c male mice subcutaneously injected with 120 mg/kg of D-gal and orally treated with 20 mg/kgof $AlCl_3$ once a day for 8 weeks.	After-treated with 1, 10, 20, 50, 100 and 150mg/kg/d for 6weeks<br>Pre-treated with 500 μg/mL for 48 h.<br>Starting from the fifth week, 25 and 100 mg/kg/day for four weeks.	↑Spatial learning capability, cognitive function<br>↓Oxidative stress<br>↓Deposition of Aβ1–42<br>↓MDA, ROS, SOD, caspase-3, Bax/Bcl-2<br>↑ Nrf2 pathway	[142]
32. Pleurotus ostreatus **polysaccharides (POP)**		Male Wistar rats induced by Al (200 mg/kg, i.g.) and D-gal (60 mg/kg, i.p.) for 60 days	On the 30th day of modeling, POP (400 mg/kg) i.g. for 30 days	↑Learning and memory performance<br>↓Oxidative stress<br>↓AchE, APP, Aβ, BACE-1, p-tau, and GSK3β<br>↑SOD, GSH-Px, CAT, PP2A	[143]
33. Alpinia oxyphylla **polysaccharides (AOP)**	76.66 kDa; Man, Rha, GlcA,	1 mg/kg/d scopolamine induced male Kungming (KM) mice for 23 days	Co-treated with 70 500 mg/kg/d for 23 days	↑Memory abilities<br>↓Inflammation<br>↓NO, IL-1β, PGE-2, and TNF-α in serum	[144]

	Glc, Gal, Xyl, and Ara (2.60: 1.95:6.73: 1.81: 21.08: 40.59: 43.83)	1 μg/mL LPS of BV2 cells for 24 h	0.325, 0.65, 1.30, 2.60 μM for 24 h		
**34.** ***Gynostemma pentaphyllum* polysaccharides (GP)**		1 mM MPP+ induced PC12 cells for 24 h	Pre-incubated 50μg/ml for 2 h	↓Apoptosis<br>↓Bax/Bcl-2 ratio, cytc c, caspase-3/9, cleavage of PARP<br>↓Mitochondrial apoptotic pathway	[145]
***35.* *Dictyophora indusiata* polysaccharides (DiP)**	86.8%Man, 4.5% Fucose, 3.9% Glc, 1.6% Gal, 1.2% Rha, 1.1% GlcA and 0.9% Xyl.	70 mM paraquat induced wildtype *C. elegans*	0.5, 1, 2 mg/mL until death	↑Survival rate	[146]
		10 mM paraquat induced *C. elegans* CL2166 for 24 h	1.0 mg/mL for 48 h	↓Oxidative stress<br>↓ ROS, MDA, MMP<br>↑SOD, ATP	
		C. elegans LG345	1.0 mg/mL at 20°C from L1 to L2 larvae	↑DAF-16/FOXO	
		C. elegans GR1352	1.0 mg/mL at 20°C from L1 to L3 larvae		
		C. elegans HA759	1.0 mg/ml for 72 h	↓Behavioral dysfunction (avoidance assay)	

Man,Mannose; Rha, Rhamnose; Glc, Glucose; Ara, Arabinose; Xyl, Xylose; GlcA, Glucuronic acid; GalA, Galacturonic acid; Gal, Galactose; i.g. intragastrically; i.p, intraperitoneal injection; EGF, epidermal growth factor; bFGF, basic FGF.↑ indicates increase/promotion/activation while ↓ indicates inhibition/reduction/inactivation.

References

133. Cao, S.; Du, J.; Hei, Q. Lycium barbarum polysaccharide protects against neurotoxicity via the Nrf2-HO-1 pathway. *Exp. Ther. Med.* **2017**, *14*, 4919–4927. https://doi.org/10.3892/etm.2017.5127.
134. Zhou, L.S.; Liao, W.F.; Chen, X.; Yue, H.; Li, S.J.; Ding, K. An arabinogalactan from fruits of *Lycium barbarum* L. inhibits production and aggregation of A beta(42). *Carbohydr. Polym.* **2018**, *195*, 643–651. https://doi.org/10.1016/j.carbpol.2018.05.022.
135. Gao, K.; Liu, M.; Cao, J.; Yao, M.; Lu, Y.; Li, J.; Zhu, X.; Yang, Z.; Wen, A. Protective effects of *Lycium barbarum* polysaccharide on 6-OHDA-induced apoptosis in PC12 cells through the ROS-NO pathway. *Molecules* **2014**, *20*, 293–308. https://doi.org/10.3390/molecules20010293.
136. Hu, Y.R.; Xing, S.; Chen, C.A.; Shen, D.; Chen, J. Codonopsis pilosula Polysaccharides Alleviate ABETA(1–40)-Induced PC12 Cells Energy Dysmetabolism via CD38/NAD+ Signaling Pathway. *Curr. Alzheimer Res.* **2021**, *18*, 208–221. https://doi.org/10.2174/1567205018666210608103831.
137. Huang, Y.C.; Tsay, H.J.; Lu, M.K.; Lin, C.H.; Yeh, C.W.; Liu, H.K.; Shiao, Y.J. Astragalus membranaceus-Polysaccharides Ameliorates Obesity, Hepatic Steatosis, Neuroinflammation and Cognition Impairment without Affecting Amyloid Deposition in Metabolically Stressed APPswe/PS1dE9 Mice. *Int. J. Mol. Sci.* **2017**, *18*, 2746. https://doi.org/10.3390/ijms18122746.
138. Li, H.F.; Shi, R.N.; Ding, F.; Wang, H.Y.; Han, W.J.; Ma, F.L.; Hu, M.H.; Ma, C.W.; Huang, Z.B. Astragalus Polysaccharide Suppresses 6-Hydroxydopamine-Induced Neurotoxicity in Caenorhabditis elegans. *Oxidative Med. Cell. Longev.* **2016**, *2016*, 4856761. https://doi.org/10.1155/2016/4856761.
139. Wang, X.L.; Yi, K.X.; Zhao, Y. Fucoidan inhibits amyloid-beta-induced toxicity in transgenic *Caenorhabditis elegans* by reducing the accumulation of amyloid-beta and decreasing the production of reactive oxygen species. *Food Funct.* **2018**, *9*, 552–560. https://doi.org/10.1039/c7fo00662d.
140. Xing, M.M.; Li, G.Y.; Liu, Y.; Yang, L.Y.; Zhang, Y.J.; Zhang, Y.R.; Ding, J.H.; Lu, M.; Yu, G.L.; Hu, G. Fucoidan from Fucus vesiculosus prevents the loss of dopaminergic neurons by alleviating mitochondrial dysfunction through targeting ATP5F1a. *Carbohydr. Polym.* , **2023**, *303*, 120470. https://doi.org/10.1016/j.carbpol.2022.120470.
141. Ma, L.J.; Wang, X.M.; Li, Y.; Xiao, H.; Yuan, F. Effect of polysaccharides from Vitis vinifera L. on NF-kappa B/I kappa B-alpha signal pathway and inflammatory factors in Alzheimer's model rats. *Biotechnol. Biotechnol. Equip.* **2018**, *32*, 1012–1020. https://doi.org/10.1080/13102818.2018.1464948.
142. Zhang, S.; Li, L.; Hu, J.; Ma, P.; Zhu, H. Taxus chinensis Polysaccharide of var. Cheng et L.K.Fu attenuates neurotoxicity and cognitive dysfunction in mice with Alzheimer's disease. *Pharm. Biol.* **2020**, *58*, 959–968. https://doi.org/10.1080/13880209.2020.1817102.
143. Zhang, Y.; Yang, X.M.; Jin, G.; Yang, X.D.; Zhang, Y. Polysaccharides from Pleurotus ostreatus alleviate cognitive impairment in a rat model of Alzheimer's disease. *Int. J. Biol. Macromol.* **2016**, *92*, 935–941. https://doi.org/10.1016/j.ijbiomac.2016.08.008.
144. Shi, W.T.; Zhong, J.; Zhang, Q.; Yan, C.Y. Structural characterization and antineuroinflammatory activity of a novel heteropolysaccharide obtained from the fruits of *Alpinia oxyphylla*. *Carbohydr. Polym.* **2020**, *229*, 115405. https://doi.org/10.1016/j.carbpol.2019.115405.
145. Deng, Q.; Yang, X. Protective effects of *Gynostemma pentaphyllum* polysaccharides on PC12 cells impaired by MPP(+). *Int. J. Biol. Macromol.* **2014**, *69*, 171–175. https://doi.org/10.1016/j.ijbiomac.2014.05.049.
146. Zhang, J.; Shi, R.; Li, H.; Xiang, Y.; Xiao, L.; Hu, M.; Ma, F.; Ma, C.; Huang, Z. Antioxidant and neuroprotective effects of *Dictyophora indusiata* polysaccharide in *Caenorhabditis elegans*. *J. Ethnopharmacol.* **2016**, *192*, 413–422. https://doi.org/10.1016/j.jep.2016.09.031.
